# Activation of the ventral tegmental area increased wakefulness in mice

**DOI:** 10.1007/s41105-017-0094-x

**Published:** 2017-02-24

**Authors:** Huan-Xin Sun, Dian-Ru Wang, Chen-Bo Ye, Zhen-Zhen Hu, Chen-Yao Wang, Zhi-Li Huang, Su-Rong Yang

**Affiliations:** 1 Department of Pharmacology, School of Basic Medical Sciences, Fudan University, 138 Yixueyuan Road, Box 229, Shanghai, 200032 China; 2 Institutes of Brain Science, Fudan University, Shanghai, 200032 China

**Keywords:** Clozapine-*N*-oxide, Pharmacogenetics, Sleep-wake, Ventral tegmental area

## Abstract

The ventral tegmental area (VTA) is crucial for brain functions, such as voluntary movement and cognition; however, the role of VTA in sleep-wake regulation when directly activated or inhibited remains unknown. In this study, we investigated the effects of activation or inhibition of VTA neurons on sleep-wake behavior using the pharmacogenetic “designer receptors exclusively activated by designer drugs (DREADD)” approach. Immunohistochemistry staining was performed to confirm the microinjection sites, and combined with electrophysiological experiments, to determine whether the VTA neurons were activated or inhibited. The hM3Dq-expressing VTA neurons were excited confirmed by clozapine-N-oxide (CNO)-driven c-Fos expression and firing in patch-clamp recordings; whereas the hM4Di-expressing VTA neurons inhibited by reduction of firing. Compared with controls, the activation of VTA neurons at 9:00 (inactive period) produced a 120.1% increase in the total wakefulness amount for 5 h, whereas NREM and REM sleep were decreased by 62.5 and 92.2%, respectively. Similarly, when VTA neurons were excited at 21:00 (active period), the total wakefulness amount increased 81.5%, while NREM and REM sleep decreased 64.6 and 93.8%, respectively, for 8 h. No difference of the amount and EEG power density of the NREM sleep was observed following the arousal effects of CNO. The inhibition of VTA neurons during active or inactive periods gave rise to no change in the time spent in the wakefulness, REM, and NREM sleep compared with control. The results indicated that VTA neurons activated pharmacogentically played important roles in promoting wakefulness.

## Introduction

The ventral tegmental area (VTA) contains dopamine (DA) containing neurons and is traversed by many blood vessels and nerve fibers. Close to its poorly defined borders are found DA and 5-HT containing neurons [1]. The VTA comprises several subdivisions and neurons synthesizing neurotransmitters, such as DA, GABA, or glutamate [2]. The DA neurons arises in the VTA and projects to: (1) the septal area, olfactory tubercle, nucleus accumbens, amygdaloid complex, and piriform cortex (mesolimbic projection) and (2) the medial prefrontal, cingulate, and entorhinal areas (mesocortical projection) [1]. The VTA is crucial for brain functions, such as voluntary movement and goal-directed behavior, as well as cognition, emotion, reward, working memory, and decision making [3–6].

Accordingly, DA midbrain system dysfunction is associated with neurologic and psychiatric diseases, such as schizophrenia, addiction, attention deficit hyperactivity disorder, and Parkinson’s disease [7]. Several lines of evidence suggest that DA signals are closely associated with regulation of sleep-wake activity [8, 9]. In humans, malfunction of the brain DA system is associated with neuropsychopathy, with disruption of sleep as one symptom [10]. Systemic administration of a selective D_1_ receptor agonist induced an increase of wakefulness (W) and a reduction of slow wave sleep (SWS) and REM sleep. On the other hand, low doses of D_2_ receptor agonist reduced W and increased SWS and REM, whereas large doses induced the opposite effect [11]. Psycho-stimulants promote wakefulness by inhibiting the DA reuptake transporter (DAT) and thereby enhancing the extracellular DA levels in mice [12]. The results are often controversial by drug effects on targets outside the VTA area or by potential disruption of neural circuits during development after gene knockout. The effects of activation or inhibition of VTA neurons on sleep/wake behavior remains to be directly tested.

The VTA is a heterogeneous area where dopaminergic and non-dopaminergic neurons are intermixed [2]. The pharmacogenetic technique “designer receptors exclusively activated by a designer drug” DREADD [13] utilizes extrinsic muscarinic receptors (hM3Dq for excitation and hM4Di for inhibition) that have lost their affinity for endogenous acetylcholine but can still be activated by a synthetic inert ligand (clozapine-*N*-oxide [CNO]). Activation of Gq-coupled hM3Dq by CNO has previously been shown to activate neurons through a phospholipase C-dependent mechanism [14]. CNO can also stimulate the Gi/o-coupled hM4Di receptor, thereby activating the inwardly rectifying potassium 3 channel, resulting in membrane hyperpolarization and neuronal silencing [13]. In this way, the activity of VTA neurons can be manipulated temporarily and reversibly. Our study established a direct link between the activity of midbrain VTA neurons and arousal promotion. The results will contribute for understanding the roles of the VTA area in regulation of sleep and wake states, which will provide a therapeutic basis for sleep disorders involved in VTA neurons.

## Materials and methods

### Animals

Pathogen-free adult male S129 mice (25–30 g) were obtained from the Laboratory Animal Center of Chinese Academy of Sciences (Shanghai, China). The animals were housed in individual cages at a constant temperature (24 ± 1 °C) with a relative humidity (60 ± 2%) on an automatically controlled 12:12 light/dark cycle (lights on at 7:00). The mice had free access to food and water. The experimental protocols were approved by the Committee on the Ethics of Animal Experiments of the University of Fudan, Shanghai Medical College (Shanghai, China). Every effort was made to minimize the number of animals used and any pain and discomfort experienced by the animals.

### Surgery

#### Virus injection

Male mice were anesthetized with chloral hydrate (5% in saline [360 mg/kg]), then placed in a stereotaxic frame, so that the head was fixed. A burr hole was made, and a fine glass pipette (1 mm glass stock, tapering slowly to a 10–20 μm tip) containing adeno-associated viral (AAV) was bilaterally lowered to the VTA (coordinates relative to bregma: anterioposterior = − 3.4 mm, mediolateral = ± 0.3 mm, dorsoventral = −4.0 mm) [15]. Then, the AAV vectors (0.05 µl per side) were injected with nitrogen gas pulses. After ten additional min, the pipette was slowly withdrawn. In our study, we employed an evolved G-protein-coupled muscarinic receptor (hM3Dq or hM4Di) that is selectively activated or inhibited by the exogenous ligand CNO. The animals recovered for 2 weeks before electrodes were implanted for electroencephalogram (EEG) and electromyogram (EMG) recordings.

#### Sleep recording and vigilance state analysis

To monitor EEG signals, two stainless steel screws were positioned 1 mm anterior to the bregma, both of which were 1.5 mm laterals to the midline [15]. Two Teflon-coated, stainless steel wires were placed into the trapezius muscles bilaterally for EMG recording. All electrodes were attached to a micro-connector and fixed to the skull with dental cement. After surgery, each mouse was allowed recover for 1 week before polygraphic recording. The recordings were started at 7:00 (onset of light period) or 19:00 (onset of dark period). We recorded EEG and EMG for two consecutive days. On day 1, the mice were treated with vehicle (intrape ritoneal [i.p.] saline) at 9:00 or 21:00, and the recordings served as the baseline data. On the next day, CNO (1 mg/kg) was injected at the same time on day 1. The same mice were used four times for different injection time and drug (9:00 saline/CNO; 21:00 saline/CNO). The interval for each experiment was 2 days.

The EEG and EMG signals were amplified and filtered (EEG, 0.5–30 Hz; EMG, 20–200 Hz), then digitized at a sampling rate of 128 Hz and recorded by VitalRecorder (Kissei Comtec, Nagano, Japan) [16]. Vigilant states were automatically classified off-line under 10-s epochs into three stages, i.e., wakefulness, rapid eye movement (REM), and non-rapid eye movement (NREM) sleep, using SLEEPSIGN (Kissei Comtec) according to standard criteria [17]. Wakefulness is defined by a low-amplitude and high-frequency EEG with a high activity of EMG. REM sleep is characterized by a low-amplitude, high-frequency EEG associated with the absence of EMG activity; the presence of EEG theta-activity (6–9 Hz) in the recording can be used to confirm this state. NREM sleep is commonly defined by a high-amplified EEG associated with a low-voltage EMG. The presence of high EEG delta activity (0.65–4 Hz) is also employed to characterize this state. As a final step, defined sleep-wake stages were examined visually and corrected if necessary [18].

### Drugs and administration

CNO (LKT Labs C4759, USA) was dissolved in saline to a concentration of 1 mg/10 ml before treatment. CNO was administered by i.p.to each mouse (0.1 ml/10 g body weight).

### Immunohistochemistry

One group of mice was injected with saline and the other with CNO at a dose of 1 mg/kg. Two hours later, the animals were deeply anesthetized with 5% chloral hydrate (360 mg/kg) and were immediately perfused transcardially with 60 ml of saline, followed by 50 ml of ice-cold 4% paraformaldehyde (PFA). The brains were removed and post-fixed for 24 h at 4 °C in 4% PFA, then equilibrated in phosphate buffer containing 30% sucrose at 4 °C. Coronal Sect. (30 μm) were cut serially on a cryostat. For double immunohistochemistry analysis, sections were thrice washed with phosphate-buffered saline (PBS) and incubated with 0.3% hydrogen peroxide in 0.1 M PBS for 30 min. After washing in PBS, the sections were incubated with a primary rabbit anti-c-Fos (1:10000, Millipore 2239640, USA) antibody diluted in PBS containing 0.3% Triton X-100 (PBST) for 48 h. On the second day, the sections were rinsed and incubated in biotinylated anti-rabbit secondary antiserum (1:1000, Jackson ImmunoResearch BA-100, West Grove, PA, USA) for 2 h. After thrice washing in PBS, all sections were then treated with avidin-biotin-peroxidase complex (1:1:1000, Vectastain ABC Elite kit; Vector Laboratories PK-6100, San Francisio, CA, USA) for 90 min. The peroxidase reactions were visualized with 0.05% 3, 3- diamino-benzidine (DAB; Sigma SK-4100, St. Louis, MO, USA) in 0.1 M PB and 0.01% hydrogen peroxide. After three washes in PBS, the sections were incubated in another primary rabbit anti-DsRed (1:10000, Takara 632496, USA) antibody diluted in PBST overnight. The procedure was similar as c-Fos staining previously described above with the exception of the last step; the color reagents of DAB did not include nickel. All sections were mounted onto gelatin-coated slides, dehydrated in graded ethanol, placed into xylene, and cover-slipped.

The sections were examined under bright-field illumination with a microscope (Leica Microsystems, Wetzlar, Germany). Using light microscopy, neurons positive for c-Fos were identified by dense black nuclear staining; the mCherry were identified by brown cytomembrane staining. Locations in the brain were confirmed by staining and reference to the primary literature and a mouse brain atlas.

### Electrophysiologic experiments

#### Preparation for brain slices

Electrophysiologic experiments were performed on slices retrieved from the mice injected with virus 3 weeks earlier. Briefly, the mice were sacrificed by decapitation, and the VTA and SNc were identified according to stereotaxic coordinates [15]. Coronal midbrain slices (300 μm thick) containing VTA and SNc were cut using a vitratome (Leica VT 1000 S, USA) in ice-cold glycerol-based artificial cerebrospinal fluid containing: glycerol (260 mM), KCL (5 mM), KH_2_PO_4_ (1.25 mM), MgSO_4_ (1.3 mM), CaCL_2_ (0.5 mM), NaHCO_3_(20 mM) and glucose (10 mM), and saturated with 95% O_2_–5% CO_2_. Slices were allowed to recover for at least 1 h in a holding chamber at a water bath (32 °C) before recording.

#### Patch-clamp recordings in the whole-cell configuration

This recording configuration was used to study the effects of CNO application on a single VTA/SNc cell. The path electrodes were pulled from borosilicate glass capillaries (1.5 mm outside diameter, 0.86 inside diameter, Sutter Instrument, Novato, CA) on a Brown-Flaming micropipette (Model P-97, Sutter Instrument, Novato, CA, USA). The path electrode had a resistance of 4–6 MΩ when filled with pipette solution containing potassium gluconate (120 mM), KCl (20 mM), MgCl2 (1 mM), CaCl2 (0.16 mM), HEPES (10 mM), EGTA (0.5 mM), MgATP (2 mM), and NaGTP (0.5 mM); with the pH was adjusted to 7.4 with KOH. A single coronal slice was transferred to the recording chamber, where the slice was held down with a platinum ring. Artificial cerebrospinal fluid (ACSF) gassed with 95% O_2_–5% CO_2_ flowed through the bath (2 ml/min). VTA/SNc neurons were identified under visual guidance using upright microscope (BX-51, Olympus, Japan) with a 40× water immersion objective lens. The cells were recorded using mCherry fluorescent signals. The image was detected with a CCD camera (U-TV1X-2, Olympus, Japan) and displayed on a monitor. The mCherry-positive neurons were clamped to record spontaneous action potentials and/or membrane potentials. The series resistance (3–5 MΩ) and input resistance (300–400 MΩ) were monitored throughout the cell recording, and data were discarded when either of the two resistances changed by >20% [19].

### Statistical analysis

All data were presented as the mean ± standard error. Histograms of the amounts of sleep and wakefulness after vehicle or CNO injection were compared using a Student’s two-tailed paired *t* test. The hourly amounts of each stage for sleep–wake profiles in mice treated with vehicle or CNO were compared using repeated-measures analysis of variance followed by Fisher’s probable least-squares difference (PLSD) test, *p* < 0.05 was considered statically significant.

## Results

### Injection sites confirmed by mCherry expression of AAV

**Fig. 1:**
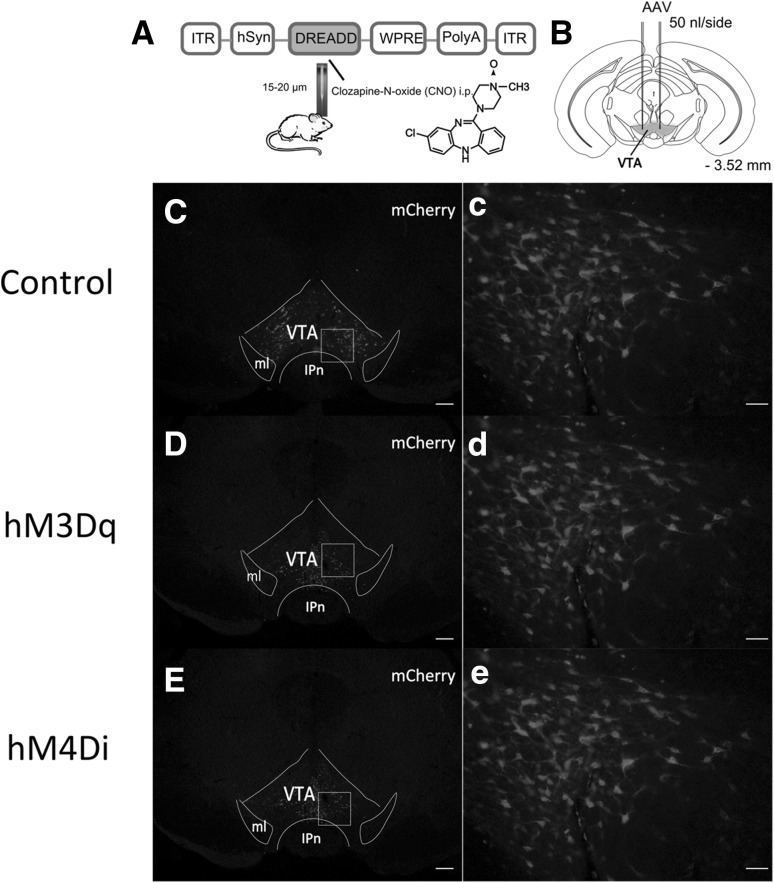
Expression of the mCherry, hM3Dq-mCherry, or hM4Di-mCherry in VTA. *A* Schematic representation of AAV vector microinjected. ITR, inverted terminal repeat; hSyn, human synapsin promoter; WPRE, woodchuck hepatitis virus post-transcriptional regulatory element. *B* Red colored sites in coronal section show the injection target in mice. *C, D, E* Coronal brain sections at the level of VTA prepared from mice expressing mCherry, hM3Dq-mCherry, or hM4Di-mCherry following hSyn-mCherry-AAV_10_ (*C*), hSyn-hM3Dq-AAV_10_ (*D*), and hSyn-hM4Di -AAV_10_ (*E*) microinjection. “*c, d, e*” higher magnification of the square region indicated in “*A, B, C*”. *Scale bars* = 100 μm (*C, D, E*); 50 μm (*c, d, e*)

To control the VTA neurons in behaving animals, we bilaterally injected the AAV containing excitatory (hSyn-hM3Dq-mCherry-AAV_10_) or inhibitory (hSyn-hM4Di-mCherry-AAV_10_) modified muscarinic G-protein-coupled receptors which can be activated by CNO into the VTA (Fig. 1AB). AAV vectors without hM3Dq or hM4Di receptors (hSyn-mCherry-AAV_10_) were used as control (Fig. 1Cc). Robust cell-surface expression of the hM3Dq (Fig. 1Dd) or hM4Dq receptors (Fig. 1Ee) was observed in the VTA.

### Modulation of the activities of VTA neurons by CNO

CNO was given by i.p. with minimal perturbation. The effects of excitatory hM3Dq and inhibitory hM4Di were examined during both the light and dark periods. After EEG/EMG recording, following treatment of CNO or saline in mice expressing mCherry, hM3Dq, or hM4Di, the animals were sacrificed and fixed 2 h later for staining. The VTA slices of these mice were examined by double staining with anti-mCherry and anti-Fos antibodies to assess the activity of these VTA neurons.

**Fig. 2:**
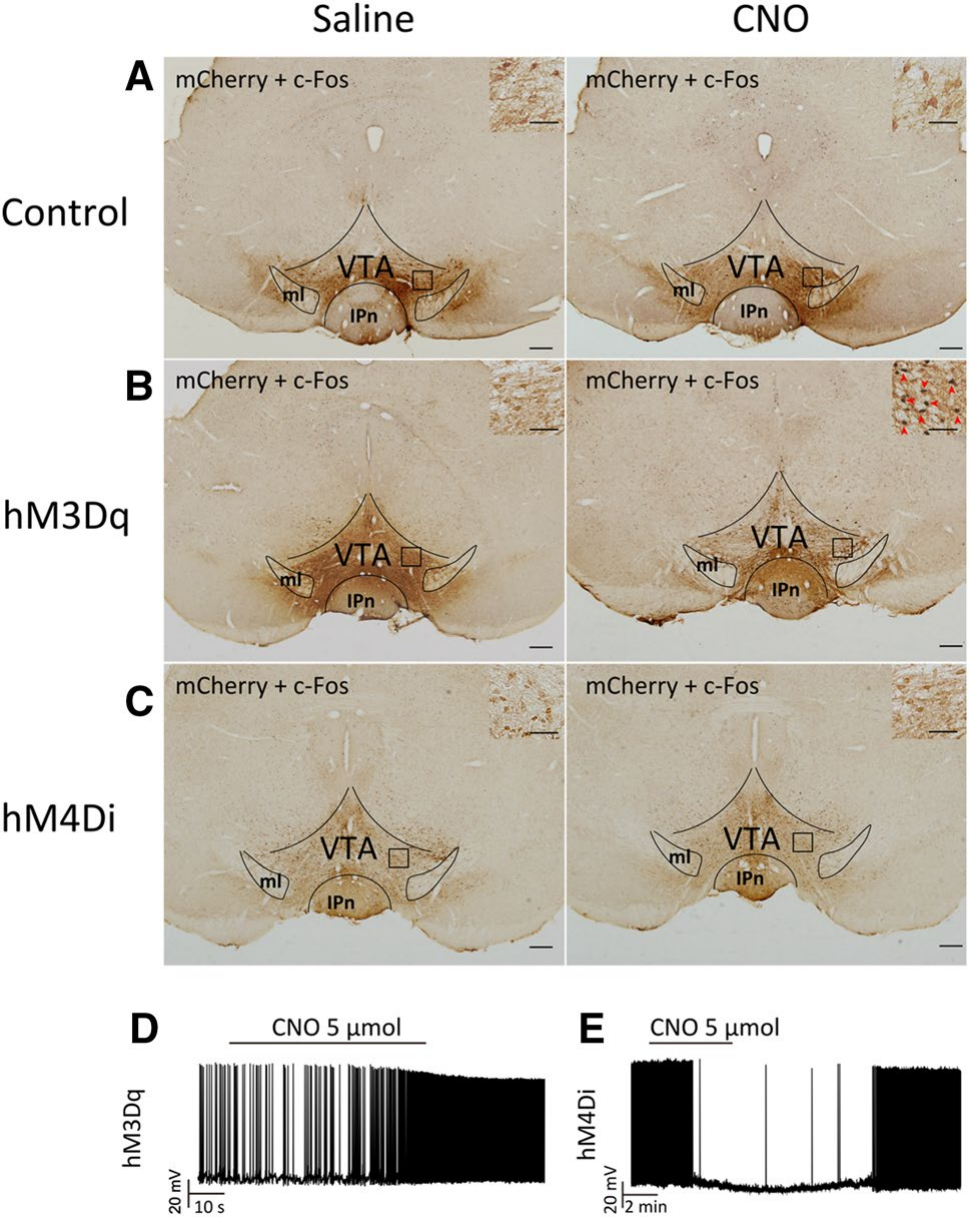
Activation or inhibition of VTA neurons by DREADD demonstrated by double staining of c-Fos and mCherry expression. **a, c** Only small numbers of c-Fos- immunoreactive nuclei were observed in mCherry-positive neurons after saline or CNO administration in mice microinjected with hSyn-mCherry-AAV_10_ (**a**) and hSyn-hM4Di-mCherry-AAV_10_ (**c**). **b** Double expression of c-Fos and mCherry (*red arrows*) induced by CNO was higher than that by saline in mice microinjected with hSyn-hM3Dq-mCherry- AAV_10_. Inset, high power view. *Scale bars* = 100 μm; *inset scales bars* = 50 μm. **d, e** Whole-cell current clamp recording showed that bath application of CNO (*horizontal bar*) produced vigorous firing of action potentials from hM3Dq/mCherry neurons (*n* = 6) (**d**) but inhibited firing from hM4Di/mCherry neurons (*n* = 6) (**e**) in the VTA. *VTA* ventral tegmental area, *ml* medial lemniscus, *IPn* interpeduncular nucleus

In wild-type mice with control AAV (hSyn-mCherry-AAV_10_) microinjection, in which VTA neurons did not express hM3Dq or hM4Di, the double-labeled neurons (mCherry-positive neurons with c-Fos-positive nuclei) were not seen (Fig. 2a). There was an apparent increase in the number of double-labeled neurons in the mice microinjected with hSyn-hM3Dq-AAV_10_ (Fig. 2b). Because the neuron activity was inhibited by hM4Di, the mice microinjected with hSyn-hM4Di-AAV_10_ showed no expression of the double-labeled neurons (Fig. 2c). Then, we carried out target recordings from hM3Dq/mCherry or hM4Di/mCherry neurons in brain slices to determine the effects of CNO on VTA neurons.

Whole-cell current–clamp recordings demonstrated that CNO (5 μmol/l) elicited vigorous firing of action potentials (Fig. 2d) in hM3Di/mCherry-positive neurons, but inhibited firing of VTA hM4Di/mCherry neurons (Fig. 2e).

### Modulation of activities of VTA neurons altered behavioral states in mice

**Fig. 3:**
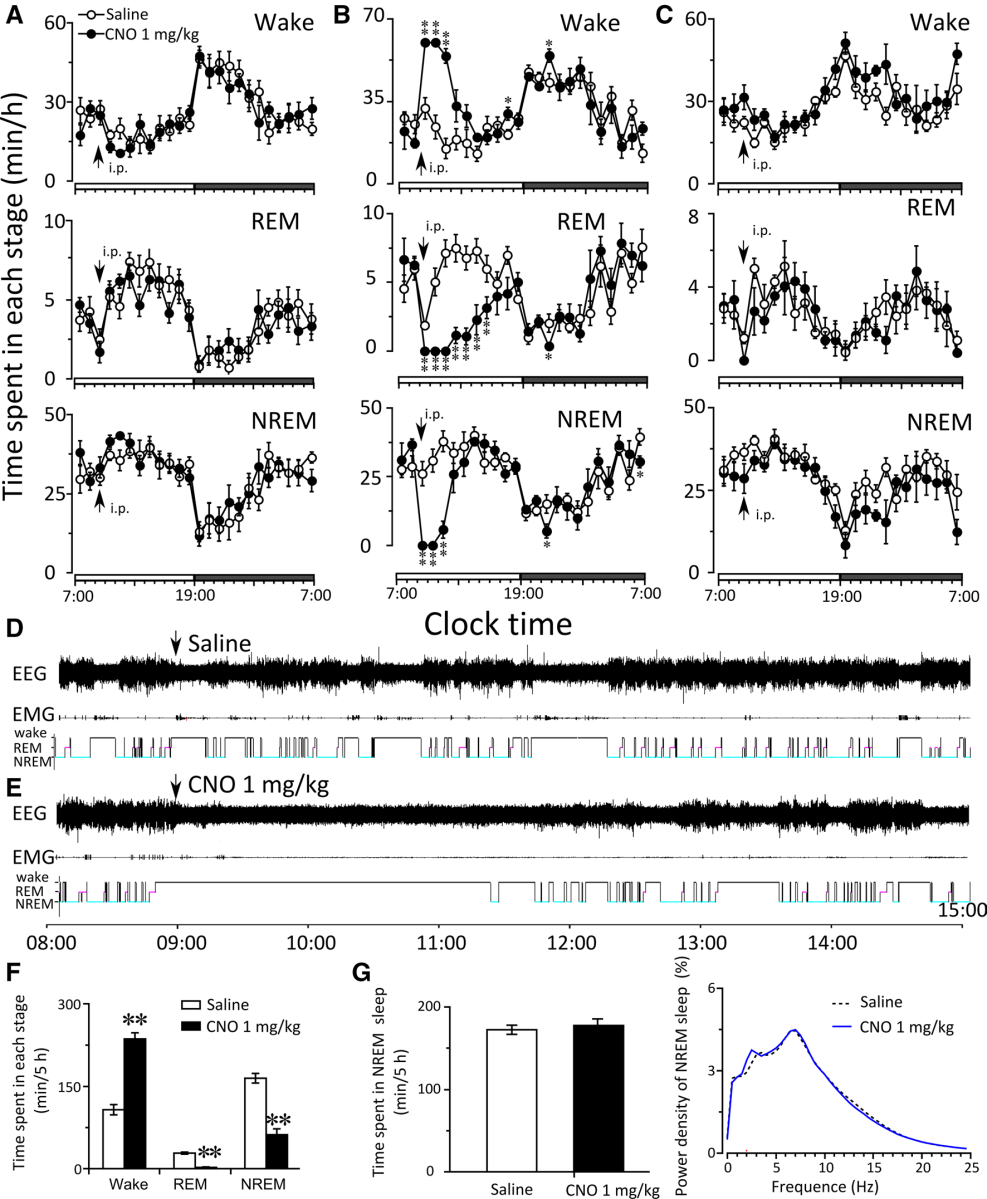
Effects of modulation of VTA neurons’ activity by DREADD during light period on vigilant states of mice. **a**–**c** Hourly amount of wakefulness, REM, and NREM sleep after CNO administration in the mice microinjected with hSyn-mCherry-AAV_10_ (*n* = 6) (**a**), hSyn-hM3Dq-AAV_10_ (*n* = 6) (**b**), and hSyn- hM4Di -AAV_10_ (*n* = 5) (**c**) into the VTA. *Each circle* represents the hourly mean ± SEM of each stage. The *horizontal open* and *filled bars* on the X-axes indicate the 12 h light and 12 h dark period, respectively. **d**–**g** In mice microinjected with hSyn-hM3Dq-AAV_10_, examples of polygraphic recording and corresponding hypnogram after treatment with saline (**d**) and CNO (**e**); time spent in each stage in 5 h (9:00–14:00) following saline or CNO injection (**f**); amount and EEG power density of NREM sleep in 5 h (14:00–19:00) following CNO-induced arousal (**g**). *p < 0.05, **p < 0.01, assessed by two-tailed paired Student’s *t* test

**Fig. 4:**
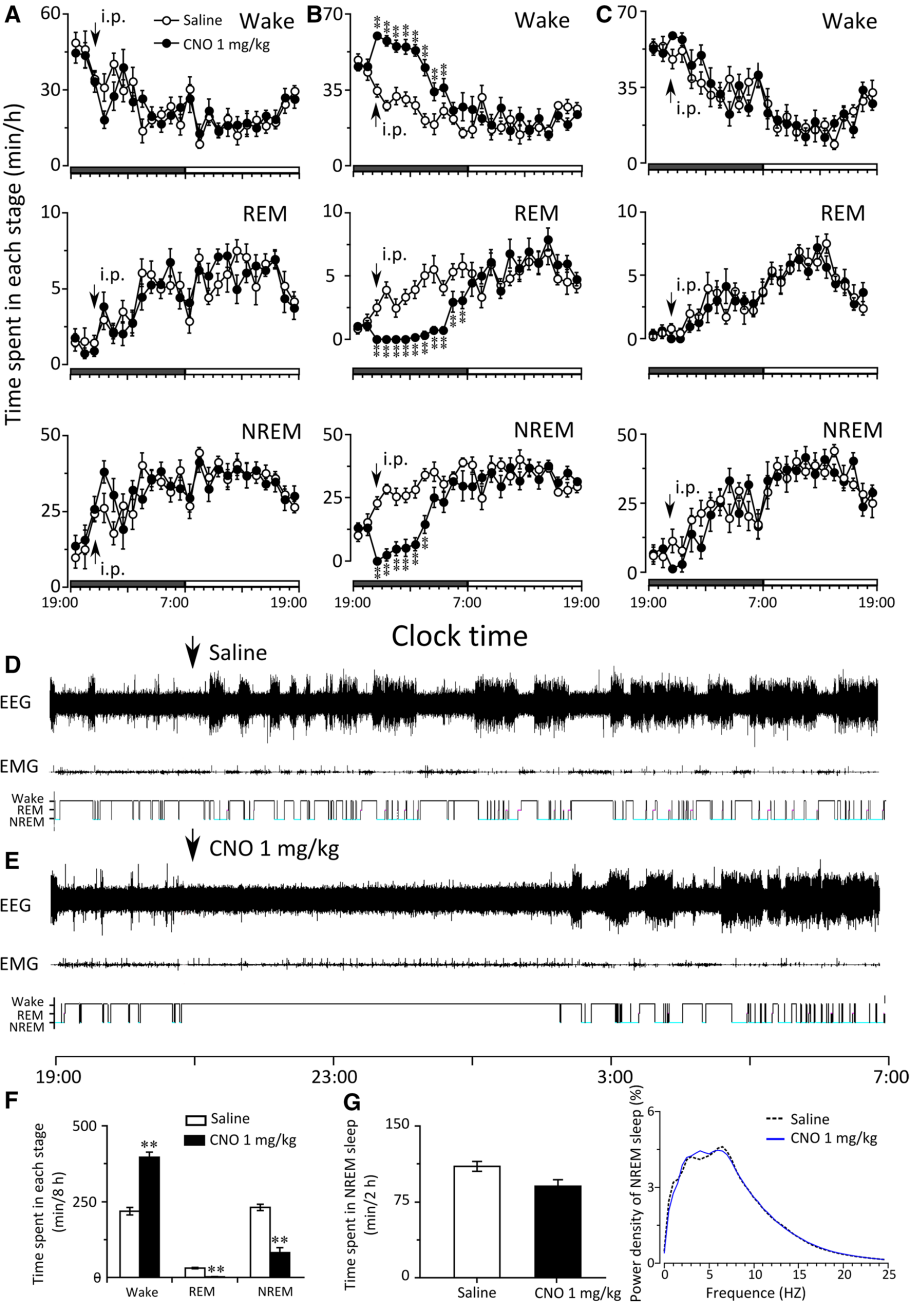
Effects of modulation of VTA neurons by DREADD’ activity during dark period on vigilant states of mice. **a**–**c** Hourly amount of wakefulness, REM, and NREM sleep after CNO administration in the mice microinjected with hSyn-mCherry-AAV_10_ (*n* = 6) (**a**), hSyn-hM3Dq- AAV_10_ (*n* = 6) (**b**), and hSyn- hM4Di-AAV_10_ (*n* = 5) (**c**) into the VTA. *Each circle* represents the hourly mean ± SEM of each stage. The *horizontal open* and *filled bars* on the x-axes indicate the 12 h light and 12 h dark periods, respectively. **d**–**g** In mice microinjected with hSyn-hM3Dq-AAV_10_, examples of polygraphic recording and corresponding hypnogram after treatment with saline (**d**) and CNO (**e**); time spent in each stage in 8 h (21:00–5:00) following saline and CNO injection (**f**); amount and EEG power density of NREM sleep in 2 h (5:00–7:00) following CNO-induced arousal (**g**). *p < 0.05, **p < 0.01, assessed by two-tailed paired Student’s *t* test

After confirming the effectiveness of the pharmacogenetic approach, we tested whether or not the selective activation or inhibition of VTA neurons affects sleep-wake states. When CNO was given at 9:00 or 21:00, the amount of wakefulness, REM, and NREM sleep in mice microinjected hSyn-mCherry-AAV_10_ or hSyn-hM4Di-AAV_10_ did not change compared with the control group (Figs. 3a, c, 4a, c).

However, the mice microinjected with hSyn-hM3Dq- AAV_10_ showed a remarkable increase in wakefulness accompanied by reduction in REM and NREM sleep after CNO administration (Fig. 3b, d, e). The sum of wakefulness in 5 h following CNO injection on light period increased 144.9% (174.2 ± 3.1 versus 71.1 ± 8.9 min, *p* < 0.01); meanwhile, the sum of REM sleep decreased 100% (0 versus 13.9 ± 1.5 min, *p* < 0.01), and NREM sleep decreased 94.0% (5.8 ± 3.1 versus 94.8 ± 7.9 min, *p* < 0.01) (Fig. 3f). When CNO was administered at 21:00 (active period), a similar phenomenon was noted in the mice microinjected with hSyn-hM3Dq- AAV_10_ as the wakefulness increased, while REM and NREM sleep decreased (Fig. 4b, d, e). The sum of wakefulness in 8 h following CNO injection increased 81.5% (396.3 ± 16.8 versus 218.4 ± 12.4 min, *p* < 0.01), meanwhile, the sum of REM sleep decreased 93.8% (1.9 ± 0.9 versus 30.8 ± 2.4 min, p < 0.01) and NREM sleep decreased 64.6% (81.8 ± 16.3 versus 230.9 ± 10.4 min, *p* < 0.01) (Fig. 4f).

Next, we analyze the EEG power density of NREM sleep after the VTA neurons’ activities were modified. In the mice microinjected with AAV-hM4Di or AAV- control, EEG power density of NREM sleep did not change (data were not shown). As for the hM3Dq positive mice, although the activation of the VTA neurons by CNO injection at 9:00 (Fig. 3g) or 21:00 (Fig. 4g) increased remarkable wakefulness, but no difference of the amount and EEG power density of the NREM sleep was observed during the time period following the arousal effects of CNO.

## Discussion

The first occurrence of the VTA in the literature is due to Tsai in 1925 [20]. In its description of the opossum brain, he identified with Nissl and Golgi staining a region lateral to the interpeduncular nucleus as the trigonum interpeduncular. Recently, the VTA DA neurons were thought to be homogeneous in their properties and behavioral functions, such that they express characteristic phasic excitatory responses to rewards and cue that predict rewards while being inhibited by omission of rewards [21]. A prior study wherein the catecholamine-containing neurons in mesencephalon were given electrolytic lesion in cats and the animals had an obvious decrease in behavioral waking indicated the catecholamine-containing neurons played a role in sleep/wake regulation [22]. However, this method produced irreversible death of neurons and may also lead to some compensatory reactions of other brain areas. Despite higher dopamine levels usually associated with arousal [12, 23], the previous studies did not establish a direct link between the activity of VTA neurons and wakefulness.

In the present study, we applied DREADD to pharmacogenetically manipulate the activity of VTA neurons. DREADD utilizes G-protein-coupled receptor signaling, which can affect neuronal activity in a relatively chronic and consistent manner and allow investigation of the behavioral effects of activation or inhibition of VTA neurons. Besides, the AAV has proven to be very effective for studying neuronal function and behavior, it is long-lived, highly neurotropic, non-pathogenic and, importantly, it can drive transgene expression at significantly high levels necessary to alter neuronal function and produce a behavioral phenotype [24]. In this study, we confirmed its effect by in vitro electrophysiological experiments, in which the spontaneous firing rates of the hM3Dq-positive neurons were increased obviously, while the hM4Di-positive neurons were inhibited by CNO administration. In this way, we can selectively manipulate the neurons in the VTA acutely and reversibly.

Considering that the firing activity of DA neurons peaked between 07:00 and 11:00 and between 19:00 and 23:00 [1], we examined the effects of the pharmacogenetic activation of the VTA neurons either at 9:00 or at 21:00 on sleep-wake behaviors in mice. We found no matter the level of sleep pressure was high or low [25], and the activation of VTA produced remarkable increase in wakefulness, which lasted longer when CNO was given during the active period in mice. The findings demonstrate for the first time that injection of CNO in mice expressing hM3Dq in the VTA is able to significantly increase arousal. Our results were consistent with previous reports that DAT knockout mice exhibited an increase in wakefulness [23], selectively activating midbrain dopaminergic neurons using DREADD induced hyperactivity in DAT-Cre mice [6], and optogenetic stimulation of VTA dopaminergic neurons initiated and maintained wakefulness in TH-Cre mice [26].

Although the VTA is generally known as the source of dopaminergic projection neurons, the VTA contains multiple cell types: dopaminergic (about 60%); GABAergic (about 35%); and glutamatergic neurons (about 5%) [2, 27]. Therefore, we think that all types of VTA neurons were simultaneously activated by this nonspecific pharmacogenetic DREADD system. VTA DA neurons project heavily to several limbic structures, including the nucleus accumbens, amygdala, and prefrontal cortex. In the rat, both GABAergic and glutamatergic neurons form local synapses in the VTA [28, 29] and project in parallel with the DA neurons to limbic regions [30, 31]. As a result, the increased wakefulness induced by activation of VTA may be caused not only by activated dopamine neurons but also by glutamatergic neurons. It may increase the dopamine and glutamate levels in targeted area which may also contribute to the wakefulness.

It is recognized that sleep is under the control of circadian and homeostatic processes, and animals will attempt to regain or compensate for sleep that was previously depleted [32, 33]. Previous studies showed that sleep deprivation often accompanied with the increase of EEG power density of NREM [34, 35]. Our results showed that the activation of VTA no matter during the day or night is responsible for wakefulness. However, the NREM sleep amount or delta power in mice expressing hM3Dq receptors treated with CNO following the induced extended wakefulness did not increase. The possible reasons may be involved the different methods of sleep deprivation, in which gentle handling or water platform techniques were employed in the previous studies, whereas the arousal was induced by VTA activation using DREADD approach. The present phenomena was in agreement with Qiu’s study, in which they did not find EEG or behavioral sleep rebound even after 4 days of induced wakefulness by chemogenetic stimulation of the pontine parabrachial nucleus [36].

To investigate whether the VTA has some roles in physiological sleep regulation, the sleep analysis in mice with hSyn-hM4Di-AAV_10_ was carried out. The inhibition of VTA neurons was confirmed with patch-clamp recording, in which CNO bath application decreased the firing of the mCherry-hM4Di positive neurons. We found there were no differences in the mice microinjected with hSyn-hM4Di-AAV_10_ between CNO and saline group in sleep-wake behavior. The reason may be a comprehensive effect by inhibition of different type neurons in VTA. When the VTA nucleus was inhibited, on one hand, the dopaminergic neurons were inhibited; on the other hand, the inhibition of the GABAergic neurons could produce disinhibition of local dopaminergic neurons. However, we cannot exclude some roles of single cell type neurons in VTA in sleep-wake regulation under physiological conditions. In fact, a recent study has shown the VTA dopaminergic neurons are necessary for wakefulness since the inhibition of these neurons suppressed wakefulness in TH-Cre mice [26].

In this study, we investigated the role of the VTA in sleep/wake regulation; however, viruses indiscriminately transduce most cell types within the introduced region. Next, we will manipulate neuronal subtypes independently to understand the genetic basis of the sleep-wake phenomena for therapeutic purposes.
